# Effective antibodies immobilization and functionalized nanoparticles in a quartz-crystal microbalance-based immunosensor for the detection of parathion

**DOI:** 10.1371/journal.pone.0171754

**Published:** 2017-02-09

**Authors:** Bartolomeo Della Ventura, Marco Iannaccone, Riccardo Funari, Massimo Pica Ciamarra, Carlo Altucci, Rosanna Capparelli, Sante Roperto, Raffaele Velotta

**Affiliations:** 1 Department of Physics “Ettore Pancini”, University of Naples “Federico II”, Naples, Italy; 2 Department of Agriculture, University of Naples “Federico II”, Portici, Italy; 3 Division of Physics and Applied Physics, School of Mathematical Sciences, Nanyang Technological University, Singapore, Singapore; 4 CNR-SPIN, University of Naples “Federico II”, Naples, Italy; VIT University, INDIA

## Abstract

**Background:**

Biosensor-based detection provides a rapid and low-cost alternative to conventional analytical methods for revealing the presence of the contaminants in water as well as solid matrices. Although important to be detected, small analytes (few hundreds of Daltons) are an issue in biosensing since the signal they induce in the transducer, and specifically in a Quartz-Crystal Microbalance, is undetectable. A pesticide like parathion (M = 292 Da) is a typical example of contaminant for which a signal amplification procedure is desirable.

**Methods/Findings:**

The ballasting of the analyte by gold nanoparticles has been already applied to heavy target as proteins or bacteria to improve the limit of detection. In this paper, we extend the application of such a method to small analytes by showing that once the working surface of a Quartz-Crystal Microbalance (QCM) has been properly functionalized, a limit of detection lower than 1 ppb is reached for parathion. The effective surface functionalization is achieved by immobilizing antibodies upright oriented on the QCM gold surface by a simple photochemical technique (Photonic Immobilization Technique, PIT) based on the UV irradiation of the antibodies, whereas a simple protocol provided by the manufacturer is applied to functionalize the gold nanoparticles. Thus, in a non-competitive approach, the small analyte is made detectable by weighing it down through a “sandwich protocol” with a second antibody tethered to heavy gold nanoparticles. The immunosensor has been proved to be effective against the parathion while showing no cross reaction when a mixture of compounds very similar to parathion is analyzed.

**Conclusion/Significance:**

The immunosensor described in this paper can be easily applied to any small molecule for which polyclonal antibodies are available since both the functionalization procedure of the QCM probe surface and gold nanoparticle can be applied to any IgG, thereby making our device of general application in terms of target analyte.

## Introduction

The detection of small molecules having a mass of a few hundreds of Dalton is of paramount importance in a variety of applications, since species like steroids, herbicides, pesticides, toxins and combustion products (e.g. polycyclic aromatic hydrocarbons) fall in this mass range and all of them are potentially harmful for human health [1–3]. The small mass of the analyte is an issue for several transducers such as quartz-crystal microbalance (QCM) [4] and surface plasmon resonance (SPR) [5] both relying on a signal which would benefit from the increase of the effective interaction area as well as from ballasting the small molecule. In the context of piezoelectric devices, gold nanoparticles (Au-NPs) have been deposited onto a pristine gold surface so to increase the effective sensing area of the electrodes [6–8].

Far more common is the use of Au-NPs to make additional links with analytes so that the apparent mass of the latter increases and an amplified response of a biosensor is achieved [9]. For instance, the QCM signal amplification through ballasting led to a reduction of the detection limit from 10.9 μg/mL to 3.5 ng/mL in an experiment where (ballasted) human IgG was detected by goat anti-human IgG [10]. Limit of detection (LOD) of 20 ng/mL has been claimed in the detection of CRP (C-reactive protein) by a QCM immunosensors using secondary antibodies conjugated with Au-NPs of 20 nm in diameter[11]. In this case a complex procedure coupled more than one Au-NP on the same secondary antibody. Other examples of QCM signal enhancement based on Au-NPs concern the detection of bacteria like *Salmonella typhimurium* [12], *Bacillus anthracis* [13] and *Escherichia coli* [14] or virus like H5N1 (avian influence) [15]. The mass enhancement can be provided even by magnetic beads, which offer the additional practical advantage of making purification easier when protein detection in real samples is faced [16].

All the above instances address the issue of improving the LOD when detecting quite heavy analytes (large proteins, bacteria or viruses). When small molecules are taken into account, the strategy to enhance their detection by adopting Au-NPs is quite spread in biosensing by SPR [17–19], electrochemical methods [20,21] or even by molecular biology assays (PCR) [22]. On the opposite, not so many examples of small molecules detection enhanced by Au-NPs can be found when QCM-based sensing is considered, one example being the detection of the aflatoxin B_1_ in contaminated milk with a LOD of 0.01 ng/mL [23]. However, this result was achieved through a complex strategy, which involved the immobilization of the complex BSA-AFB_1_ on the QCM, a subsequent blockage by BSA solution, and finally the competitive immunoreaction between AFB_1_ on the probe surface and that in the sample with monoclonal anti-AFB_1_ antibody. A similar competitive approach was adopted in the detection of adenosine by a QCM with dissipation measurement, which was functionalized by adenosine aptamer sequence [24]. In this scheme the absence of adenosine allowed the aptamer immobilized onto the chip surface to hybridize with the random coiled detection part carrying Au-NPs (high frequency shift), whereas the presence of adenosine led to a complex structure of the aptamer preventing it from binding the reporter part (low frequency shift); thus, the lower the adenosine concentration the larger the frequency change. The limit of detection reported in this paper is 65 nM, which translates into 17 ng/mL when the molecular weight of adenosine is taken into account. In spite of its several drawbacks, the competitive approach is sometimes considered the only option when small molecules have to be detected even if non-competitive approach is always desirable [25].

Since QCM has several advantages and its adoption is beneficial in many circumstances [4,26], it is timely to improve its sensitivity so to extend its application to the detection of low concentration of small molecules without resorting to competitive assay. In this respect, although necessary, the ballasting is not enough to warrant success when the detection of small molecules is addressed since the signal enhancement can be hampered by a surface not properly functionalized. In fact, it is vital to choose an immobilization method that not only warrants an effective surface covering [27,28], i.e. the number of Abs per surface unit, but also their orientation [29]. This is because an uniform orientation of the recognition elements provides a huge systematic improvement in sensitivity for weak interactions and such an effect is even more pronounced for smaller molecule for which the number of epitopes per analyte is necessarily low [30].

In a number of previous papers, we have shown the effectiveness of the Photonic Immobilization Technique (PIT), which is able to accomplish a gold surface fully covered by oriented antibodies [31–33]. Thus, motivated by the quest for a simpler, non-competitive assay, we realized a QCM-based immunosensor joining an effective functionalization procedure, i.e. PIT, to a ballasting technique based on commercial Au-NPs subsequently functionalized with Abs. As a case study, we chose the parathion (IUPAC name O,O-diethyl O-4-nitrophenyl phosphorothioate), which has a low molecular weight (292 Da) and high interest for environment and health safety. Parathion is a pesticide widely used to enhance agricultural production, but for its toxicity [34] it is now forbidden within the European Union which sets the limits of pesticide residues in food between 20 and 50 μg/kg (Commission Regulation (EC) No 839/2008). A biosensor-based detection allowing *in situ* and real-time analysis for environmental monitoring and food quality control would offer many advantages compared to complex techniques like High-Performance-Liquid-Chromatography (HPLC) and/or mass spectrometry. In our previous papers, we achieved LODs for parathion of 60 μg/L and 15 μg/L by ballasting the analyte with Bovine Serum Albumin (67 kDa) [32] and antibody (150 kDa) [33], respectively.

In the present study, we report on the realization of a QCM-based immunosensor able to reach a LOD as low as 0.8 μg/L (<1 ppb) in a non-competitive scheme. The immunosensor is functionalized in an effective way through the PIT, so that anti-parathion polyclonal antibodies upright oriented fully cover the probe surface, and the signal is enhanced by ballasting the parathion in a sandwich configuration with the same anti-parathion polyclonal Abs themselves tethered to Au-NPs. The commercial amino coated Au-NPs are functionalized with polyclonal Abs against parathion through a simple procedure requiring only few minutes. The immunosensor shows an excellent specificity even against a molecule like paraoxon, which differs by parathion in just one atom. We also show that the enhanced signal can be explained by assuming that the probe surface uptakes nanoparticles via random sequential adsorption process and that the saturation of the signal corresponds to a surface fully covered by functionalized gold nanoparticles.

## Materials and methods

### Chemicals

Parathion (45607) was purchased from Sigma-Aldrich and anti-parathion (ABIN113883) polyclonal antibodies was purchased as rabbit sera from Antibodies-online.com. Au-NPs 10nm OD20 were purchased from InnovaCoat^®^. The type G immunoglobulins (IgG) were purified using the Protein A Antibody Purification Kit (PURE1A) from Sigma-Aldrich. 5,5′-dithiobis-(2-nitrobenzoic acid) also known as Ellman’s reagent (D8130), Bovine serum albumin (A2153) and the compounds used for the specificity tests, p-nonylphenol (46018), dichlorvos (45441), diazinon (45428) and paraoxon (36186), were from Sigma-Aldrich. The pollutant samples were prepared using PBS 1x buffer solution in the fume hood. MilliQ water, sulfuric acid 98% and hydrogen peroxide 40% were used for the cleaning procedure of the QCM gold surfaces.

### Gold nanoparticles and their functionalization

The requirement of ballasting the secondary Ab with heavy nanoparticles inherently leads to the choice of gold as material in view of its high density (*ρ*_Au_ = 19.3 g/cm^3^). As it concerns the diameter, in order to keep for the Au-NPs the condition of Brownian motion with a constant barometric profile [35] on a length of the order 1 mm (the height of the cell above the quartz is approximately 0.3 mm), we chose a diameter of 10 nm. The Au-NPs were purchased from InnovaCoat^®^. To functionalize the nanoparticles with antibodies, we followed the protocol suggested by the company, which includes the following steps:

1. Preparation of stock of antibodies against parathion with concentration of 0.25 mg/mL.
2. Mixing of 12μL of the antibody stock and 42μL of reaction buffer in a microfuge tube (room temperature).
3. After 15 minutes wait, addition of 5μL quencher to stop the reaction, and gentle mixing.
4. After 5 minutes wait, washing the particles by adding 450μL of PBS 1x and centrifugation at 21500g for 45 minute.

**Fig 1:**
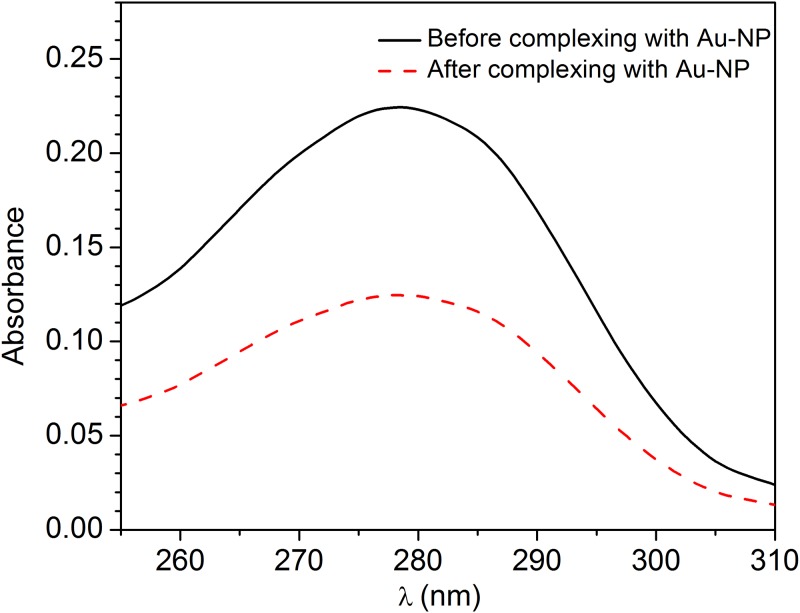
Absorption spectra of a solution containing 180 μg/mL of polyclonal antibody against parathion measured before (continuous black line) and after the functionalization of the Au-NPs (dashed red line).

In order to evaluate the mean number of Abs per Au-NP, we assessed the antibody concentration before and after the Au-NP functionalization procedure and considered the missing fraction as complexed with the Au-NPs. To this end we measured the absorbance of an initial solution containing 180 μg/mL of polyclonal Ab against parathion, which corresponds approximately to a number of antibodies 7×10^14^ Abs/mL.[36]. The result is reported in Fig 1 (continuous black line) where the typical absorption peak of proteins at 278 nm is visible. After the incubation of Abs with a solution of 1.14×10^14^ Au-NPs/mL, we recovered the free antibodies in the supernatant after centrifugation (9000 rpm for 5 minutes) while Au-NPs complexed with Abs were sedimented into the pellet. The absorbance of the supernatant is reported in Fig 1 (dashed red line) showing a reduction of the Ab concentration of approximately 45%, which corresponds to a number of antibodies complexed with Au-NPs of 3.2×10^14^ Abs/mL. Since we have 1.14×10^14^ Au-NPs/mL, the mean number <n> of Abs per Au-NP is

$$\langle n\rangle \approx \frac{3.2\times 10^{14}\text{ Abs}/\text{mL}}{1.14\times 10^{14}\text{ Au}-\text{Nps}/\text{mL}}\approx 3\frac{\text{Abs}}{\text{Au}-\text{Np}}$$ (1)

### UV laser source

The immunoglobulin samples were irradiated using the UV laser pulses provided by a custom femtosecond PHAROS laser system with high tunable pulse repetition rate coupled with a harmonic generator stage (HIRO) which allows the conversion to 515 nm, 343 nm and 258 nm wavelengths of the IR fundamental radiation. Both PHAROS and HIRO were from Light Conversion Ltd.

### QCM setup

The quartz oscillators (151218) are from ICM, Oklahoma city (USA). They are AT-CUT quartz with a fundamental frequency of 10 MHz. The crystal and the gold electrode diameters are 1.37 cm and 0.68 cm respectively. The gold surfaces are cleaned by immersing the oscillators for 1 min in a glass beaker containing Piranha solution (1:1 ratio between concentrated sulfuric acid and 40% hydrogen peroxide solution), then the quartzes are washed with helix water. The whole cleaning procedure is performed in the hood.

The QCM device (open source QCM) is purchased from Novaetech, Italy (http://openqcm.com/) and oscillates at a frequency *f*_0_ = 10 MHz. The gold-quartz wafer is placed on the electronic console and the resonance frequency of the oscillator is monitored by producer released software. The QCM is integrated in a fluidic circuit consisting of the cell which contains the oscillator, tygon tubes and a GILSON peristaltic pump. The volume of the circuit is about 300 μL, the flow rate is 3 μL/s and the volume of the liquid interacting with the QCM (i.e. the cell) is approximately 10 μL. The response of the QCM is proportional to the mass tethered to the electrode [37]

$$\Delta f=\frac{-2f_{0}}{A\sqrt{\rho_{q}\mu_{q}}}\Delta m$$ (2)

where Δ*f* is the frequency shift, *f*_0_ the resonance frequency, *A* the piezoelectric active area, *ρ*_q_ the density of quartz, *μ*_q_ the shear modulus of AT-cut quartz crystal, and Δ*m* the deposited mass. By considering the actual values for quartz and the area of the active probe surface *A* = 0.2 cm^2^, we have Δ*f*[Hz]≈-Δ*m*[ng].

### UV activation of antibody solution

The immobilization of the antibodies onto the gold surface is realized by the Photonic Immobilization Technique (PIT) [31]. This technique relies on the photonic reduction of disulfide bridges in proteins by UV illumination of near aromatic amino acid [38] and leads to antibodies to expose the fragment antigen binding (Fab) onto thiol-reactive surfaces like gold plates [39]. To apply this technique it is necessary the protein has a closely spaced tryptophan/cysteine-cysteine (Trp/Cys-Cys) triad, which is a typical structural characteristic of the immunoglobulin family [40]. Basically. the UV-excitation of tryptophan can result in its photoionization which generates a solvated electron that is captured by the near electrophilic cysteine [41]. This results in the breakage of the disulphide bridge, whereby a thiol is formed which can easily react with thiol reactive surfaces like gold electrodes. The rise of the number of the SH groups onto the protein allows new structural conformation for the immobilized immunoglobulin which are characterized by a well exposure of the antigen binding sites thus greatly improving sensor sensitivity [39]. It is well known that UV radiation strongly affects both structure and activity of biomolecules, but we have recently demonstrated that the photonic activation of immunoglobulins does not affect their ability to capture the antigen [32].

To realize the PIT, samples of 500 μL with an antibody concentration of 25 μg/mL were activated using the UV laser source previously described. The best irradiation conditions to maximize the number of thiol groups per molecule are λ = 258 nm, 10 kHz repetition rate, 250 mW of average power, and 1 minute irradiation time.

## Results and discussion

### Detection scheme and QCM sensorgram

**Fig 2:**
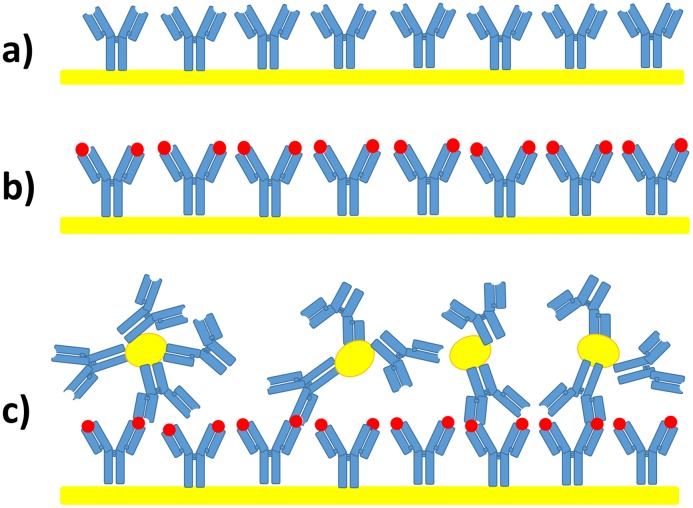
Schematic principle of the measurement procedure. a) The irradiated Abs are tethered right-up onto the gold electrode, thereby being able to recognize the analyte with large effectiveness [panel b)]. c) The detection of the light analyte takes place by ballasting it in a specific way through secondary Abs linked to Au-NP.

The strategy adopted to enhance the sensitivity of the QCM-based immunosensors is sketched in Fig 2(a)–2(c). Thanks to PIT the Abs are covalently bound to the QCM gold surface with antigen binding site exposed to the fluid (a). The solution containing the parathion is subsequently conveyed to the cell (b) and the analyte is recognized by the Abs. At this stage, no appreciable signal is detected in view of small mass of the parathion, but the following interaction with a ballast constituted by functionalized Au-NPs (c) allows one to detect the presence of small molecules. In this scheme the same Abs used to functionalize the gold surface of the QCM are tethered to the Au-NPs by the protocol described in the Materials and Methods section.

**Fig 3:**
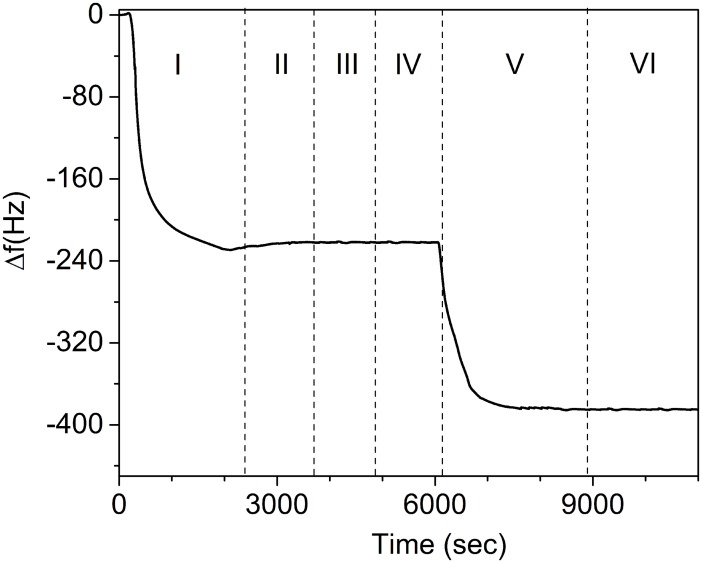
Sensorgram showing the output of the QCM, which includes the functionalization (steps I-III) and the measurement (steps IV-VI) of parathion at 290 μg/L. I: functionalization by PIT (Abs are tethered to the probe surface); II: the washing by PBS solution (1x) removes unspecific bonds giving rise to a small increase of the frequency; III: BSA is conveyed to the cell and no frequency change is observed warranting the full coverage of the surface: IV: parathion is injected, but no frequency change is observed because of its small mass; V: the injection of the Au-NPs complexed with Abs against parathion yields a huge frequency change; VI: the eventual washing with PBS (1x) does not change the frequency since all the bonds are specific.

The detailed steps followed to measure the parathion concentration lead to a sensorgram like that reported in Fig 3 achieved for a parathion solution of 290 μg/L. After the reaching of the basal frequency stabilization with PBS solution flowing in the circuit, the surface is functionalized (step I) using previously irradiated antibodies (PIT), which tether the surface providing a first frequency drop of about 230 Hz. Subsequently, a washing step (II) with PBS is used to purge the circuit from the excess of immunoglobulins. Then (III), a BSA solution (50 μg/mL) flows into the cell filling the remaining free space on the gold surface. This blocking step is crucial in order to avoid possible non-specific interactions between the further flowing molecules and the gold plate. It is worth noticing that step III results in a negligible change in the resonance frequency, therefore proving that the gold surface is quite completely covered by the antibodies. In the next step (step IV), the solution containing parathion 290 μg/L flows in the circuit and, in view of the small mass of the analyte, no significant frequency shift is produced. Finally (step V), the colloidal solution with Au-NPs (1.14×10^14^ AuNp/mL) is conveyed to the cell giving rise to the sandwich configuration sketched in Fig 2(c), which gives rise to a frequency shift of 160 Hz. The lack of frequency changes in the final wash (step VI) warrants that no significant non-specific or weakly bindings occur in our protocol.

It has to be noticed that even at parathion concentration as high as 290 μg/L no significant frequency change can be detected without ballasting the analyte. The steps I-III correspond to the functionalization, which is very well reproducible with PIT and are not influenced by the subsequent measurements, whereas the steps IV-VI relate to the actual measurement, which lasts less than 2 hours. The final frequency shift Δ*f* provides the measurement of the concentration.

### Dose-response curve (LOD)

**Fig 4:**
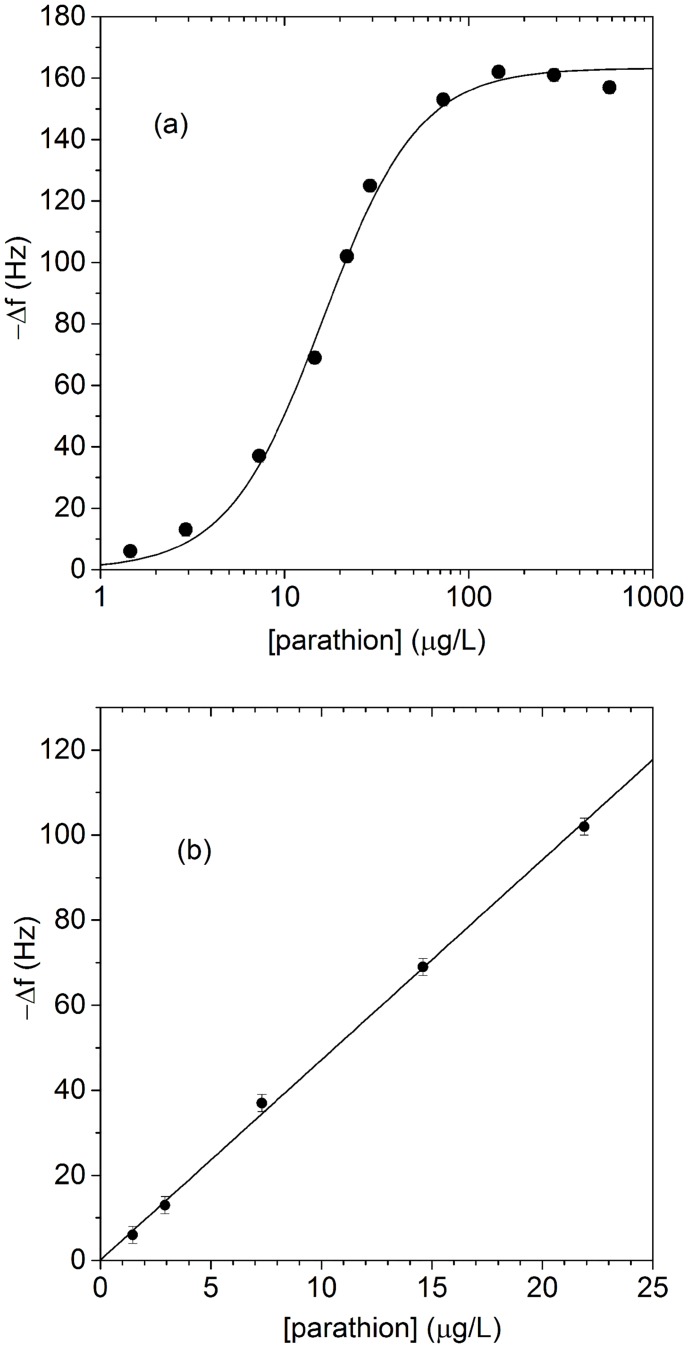
Dose response curve of the QCM-based biosensor with the ballasting protocol depicted in Fig 2. (a) The whole set of measurements reported in log scale. The error is smaller than the size of the point. (b) Magnification of the low range concentration showing an excellent linearity up to approximately 30 μg/L. The size of the points is smaller than in the panel (a) to highlight the uncertainty of ±2 Hz.

The results as a function of the parathion concentration are reported in Fig 4(a) where a saturation is observable at parathion concentration above 100 μg/L. The data have been fitted with a sigmoidal function and since they cover a range of approximately three decades, they are best viewed with logarithmic scale. The magnification of the range 0–25 μg/L is shown in Fig 4(b) where the best fit of the experimental data with the linear function

Δf=a[P]+b (3)

yields *a* = 4.7±0.1 Hz/(μg/L) for the slope and *b* = 0.1±1.2 Hz for the intercept. In view of its uncertainty, the value for *b* ensures the lack of any systematic error as it is compatible with zero.

Since the intrinsic limitation of the frequency measurement is very small, the uncertainty in our measurements is essentially due to the unavoidable fluctuations in the several steps of the measurement protocol. Nevertheless, as the results in Fig 4(b) show, we are able to keep the error on the single measurement in the range of ±2 Hz. Since the LOD is the concentration providing the lowest frequency shift significantly different from zero, we can evaluate such a parameter by inverting Eq (3) with *b* = 0 and (Δ*f*)_min_ = 4 Hz, the latter value chosen to be twice the uncertainty on the single measurement (95% confidence interval)

$$\text{LOD}\approx \frac{{\left(\Delta f\right)}_{\text{min}}}{a}\approx 0.8\;\mu \text{g/L}.$$ (4)

### Specificity test

**Fig 5:**
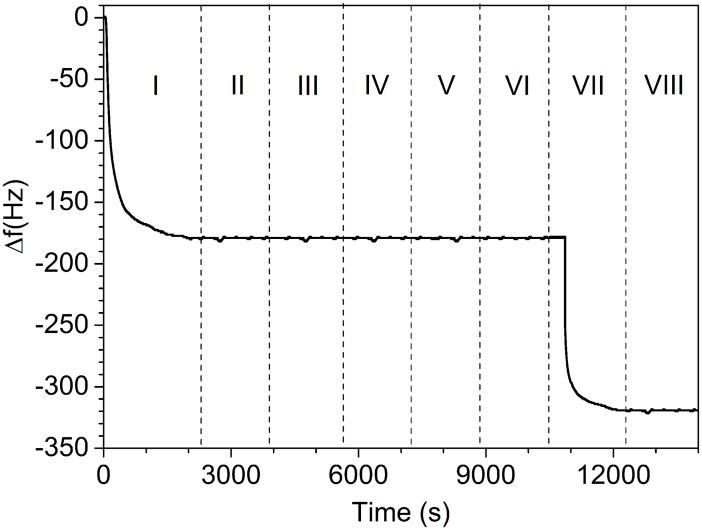
Sensorgram showing the output of the QCM to test the specificity of the biosensor. I: PIT functionalization with antiparathion; II: washing with PBS (1x); III: mixture of pollutants (SOL1) at high concentration (>200 μg/L for each of pollutant); IV: Au-NPs complexed with antiparathion; V: washing; VI: SOL2 = SOL1+parathion at a concentration of 50 μg/L; VII: Au-NPs complexed with antiparathion; VIII: washing with PBS (1x).

To ascertain the sensor specificity, we tested the response of the functionalized QCM against a molecule very similar to parathion like paraoxon (diethyl 4-nitrophenyl phosphate, *M* = 275 Da), but also against other pesticides like dichlorvos (2,2-dichlorovinyl dimethyl phosphate, M = 221 Da) and diazinon (O,O-Diethyl O-[2-isopropyl-6-methyl-4-pyrimidinyl] phosphorothioate, *M* = 304 Da) as well as against nonylphenol (4-nonylphenol, *M* = 220 Da), the latter being a precursor in pesticide production. To this end we prepared two solutions: the first solution (SOL1) with a concentration of 2 μM of all the compounds but parathion (this corresponding to a concentration >200 μg/L for these molecules) and a second solution SOL2 which contained the same concentration of pollutants as SOL1, but containing also parathion with a concentration of 50 μg/L. The result of the test is reported in Fig 5 where the step I corresponds to the functionalization and the step II to the washing (see also Fig 3).

When SOL1 flows into the QCM no response is provided by the sensor even when the Au-NPs complexed with antiparathion are conveyed to the cell (steps III and IV). This demonstrates that none of the pollutants is recognized by the antiparathion even if one of the pollutant (paraoxon) differs from parathion by just one atom (in paraoxon oxygen replaces the sulphur of parathion); moreover, the lack of any frequency change after step IV also demonstrates that the complexed Au-NPs do not interact with the functionalized gold surface unless a sandwich configuration with the parathion as a mediator is realized (see below). The frequency stability even after the subsequent washing (step V) warrants that the sensor is ready for another test (step VI), which this time is carried out with SOL2 containing parathion at a concentration of 50 μg/L and other pollutants at higher concentration (>200 μg/L). Once again, there is no change in the frequency because of the low mass of the analyte, but on this occasion when the functionalized Au-NPs are conveyed to the cell (step VII) a frequency shift of 140 Hz is observed in agreement with the dose-response curve reported in Fig 4. The final washing (step VIII) warrants that the sandwich configuration realized between immobilized antiparathion and Au-NP by means of the recognized parathion (Fig 2) is stable.

It is worth mentioning that specificity and sensitivity are two important features of a biosensor. While the former is intrinsically related to the type of biomolecule involved in the detection procedure (antibody in this case), the latter mostly depends on the orientation of the immobilized proteins. Thanks to the PIT, immunoglobulins take an effective orientation and, hence, the antigen is captured with high efficiency, thus greatly enhancing the *sensitivity* of the device. On the other hand, PIT does not affect the recognition properties of the antibodies so that the device operates with high *specificity*.

### Filling factor of QCM surface as a result of random sequential adsorption process

**Fig 6:**
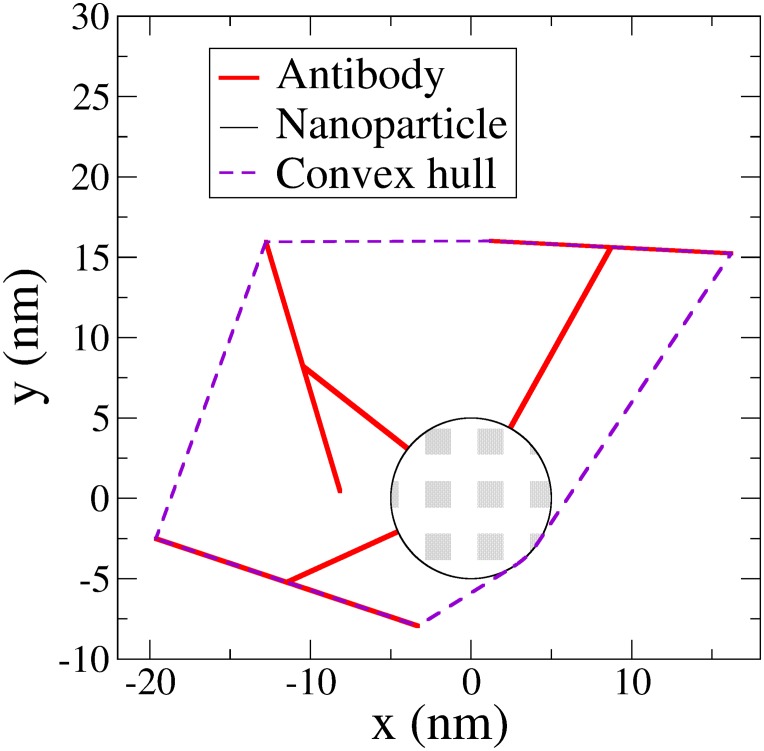
The projection of a model Au-NP functionalized with Abs consists of a circle, which is the projection of the gold nanoparticle and of different segments associated to the T shape of the antibodies. We estimate the area occupied by the functionalized particle on the sensor as that of the convex hull of these geometrical objects. The mean occupied area is obtained by averaging over different randomly functionalized nanoparticles.

As a final note, we provide an estimation of the filling fraction of the Au-NPs complexed with anti-parathion in saturation condition. The filling fraction is the area of the sensor occupied by the particles, *φ = ρS*, where *ρ* is surface density of captured nanoparticles, and *S* the average projected area of the Au-NPs complexed particles on the surface. The surface density results to be *ρ* = *N/A* = 8×10^−4^ nm^-2^, where *A* = 0.2 cm^2^ is the area of the sensor, and *N* = *M*/*m* the number of captured complexes. Here *M*≈160 ng is the total mass of recognized complexed particles in saturation condition [see Fig 4(a)] as estimated through Eq (2), while *m*≈10^−8^ ng is the mass of a single particle. A rough estimation of *S* could be carried out by assuming the projection of the particle on the surface as given by a circle with radius *R*≈20 nm, i.e. by considering the sphere circumscribing the Au-NP complexed with Abs, the latter having the linear size of approximately 15 nm. This would lead to the unrealistic filling factor *φ*≈1, which would be even larger than 0.9 corresponding to the maximum filling fraction of a collections of non-overlapping disks on a hexagonal lattice. Evidently, this is the result of the overestimation of the area pertaining to each complexed Au-NP, which is not a sphere anymore after being functionalized with Abs. Thus, for a more realistic estimation we have devised a numerical procedure to compute the projected area of a functionalized Au-NP by specifying the positions of the nanoparticle, identified by its center, and of three antibodies randomly located on the surface, each one identified specifying the two segment giving rise to its T shape (see Fig 6). The radius of the sphere and the length of the segment are chosen to reproduce the size of the nanoparticle and antibodies, respectively. The projection of this three dimensional structure on a plane consist of a circle, and of different connected segments, as shown in Fig 6. The projected area of the particle can now be estimated as corresponding to that of the convex hull of these objects.

We remind that, in two dimensions, the convex hull of a set of points is the smallest convex polygon containing all of them. By averaging over model functionalized nanoparticles differing for the position and orientation of the functionalizing T shaped molecules, that are randomly chosen, we estimate the average projected area to be S≈610 nm^2^, and thus *φ*≈0.5. This value is compatible with the theoretical prediction [42] for the filling fraction of anisotropic particles deposited on a surface through a random sequential adsorption process. The use of this model to describe the binding of functionalized Au-NPs is consistent with their size (10 nm diameter plus the length of the Abs) larger than the typical distance between the Abs deposited on the surface, which we estimate from their density to be approximately 5 nm. This implies that the whole surface is as able to bind the functionalized nanoparticles, and that, as in random sequential adsorption processes, the factor limiting their number is their steric hindrance.

## Conclusions

We report the description of a QCM-based immunosensor for detecting small molecules. The device combines the recently proposed Photonic Immobilization Technique for the antibody functionalization of the gold surfaces and the antibody-sandwich protocol. While PIT is shown to increase the sensitivity of QCM by orienting the antibodies upright with high effectiveness, the sandwich protocol has a twofold effect: on the one hand it weighs down light molecules, so that they can be “weighed” by QCM, on the other hand it inherently increases even more the specificity of the whole device. By applying for the first time the gold nanoparticle ballasting to a small molecule, we show that a LOD of 0.8 μg/L for parathion in water can be reached.
